# Raised Glycated Hemoglobin (HbA1c) Level as a Risk Factor for Myocardial Infarction in Diabetic Patients: A Hospital-Based, Cross-Sectional Study in Peshawar

**DOI:** 10.7759/cureus.25723

**Published:** 2022-06-07

**Authors:** Saud Azhar, Fahd Zafar Khan, Shahmir Tariq Khan, Bushra Iftikhar

**Affiliations:** 1 Community Medicine, Khyber Medical College, Peshawar, PAK

**Keywords:** hospital-based, cross-sectional study, cardiovascular disease, pakistan, diabetes mellitus, peshawar, fasting blood sugar, glycated hemoglobin (hba1c), uncontrolled diabetes, myocardial infarction

## Abstract

Background

Diabetes is a rapidly rising chronic illness in developing countries. The main objective of this research is to compare the frequency of myocardial infarction (MI) in controlled and uncontrolled diabetics in Pakistan, especially in the underprivileged district of Peshawar, and to determine raised blood glucose as a risk factor for MI.

Methodology

This cross-sectional study involving 237 diabetic patients aged 30-80 years was conducted in three major tertiary care hospitals in Peshawar, Pakistan. The inclusion criteria were diabetic patients with glycated hemoglobin (HbA1C) levels of less than 7% considered to be “controlled diabetics” and above 7% considered to be “uncontrolled diabetics.” Data were collected using structured questionnaires, past medical records, and patient history and were analyzed using SPSS software (IBM Corp., Armonk, NY, USA). The study was concluded in March 2022.

Results

The highest number of MIs occurred in diabetics with HbA1c levels of 8-9% (47.9% of all MIs). There was a significant association between increasing HbA1c levels and the incidence of MI (p = 0.002). The adjusted prevalence odds ratio for MI in uncontrolled diabetics was 6.105 (95% confidence interval = 2.42-15.43), that is, six times increased incidence of MI in patients with HbA1c of more than 7%. Furthermore, with a 1% increase in HbA1c, there was a 10% increase in the proportion of MIs.

Conclusions

From this study, it is clear that HbA1c levels of 8-9% were most significantly associated with the risk of MI in uncontrolled diabetics, and with rising levels of HbA1c, the risk of MI increased significantly. Thus, this study highlights the importance of HbA1c control in diabetic patients to prevent a heart attack.

## Introduction

The change in lifestyle and food consumption patterns throughout the world has led to a change in trends of disease mortalities which has shifted from infectious diseases, such as tuberculosis, pneumonia, influenza, and malaria, to chronic diseases, such as ischemic heart disease (IHD), stroke, cancers, diabetes mellitus (DM), and hypertension. In particular, DM is crucial because it leads to multiple complications in patients that lead to their untimely death if left unchecked. According to the World Health Organization, by 2020, DM would be the cause of 70% of mortality from all chronic diseases that cause death in developing countries [1]. Even more concerning is the projected 2.5-fold increase in the population of individuals with DM in the developing world from 1995 to 2025 [1].

Cardiovascular disease (CVD) is the major cause of mortality in individuals with DM, especially in uncontrolled long-standing type II DM, and DM remains the number one underlying risk factor for CVD. DM also leads to other conditions that contribute to heart diseases, such as hypertension and hypercholesterolemia. According to the American Heart Association [2], at least 68% of individuals aged 65 or older with diabetes die from some form of heart disease. Adults with diabetes are two to four times more likely to die from heart disease than adults without diabetes. The American Heart Association considers diabetes to be one of the seven major controllable risk factors for CVD. Risk factors other than DM that lead to MI must also be investigated and accounted for as they are a part of the variables and control factors in such research.

Elevated blood glucose level is the most damaging element of uncontrolled DM that may lead to IHD, the most common type of CVD. Elevated blood glucose levels cause vascular damage, especially to the susceptible coronary arteries, by accelerating atherosclerosis, endothelial cell dysfunction, glycosylation of extracellular matrix proteins, and vascular denervation [3]. Persistent vascular damage leads to inflammation, stiffening, stenosis, thrombosis, or even rupture of the coronary arteries which leads to myocardial infarction (MI). Moreover, high blood glucose levels in patients at the time of acute MI have been shown to be associated with a poor prognosis of MI (regardless of whether patients have DM or not) [4]. This also shows the negative impact of high blood glucose on the heart. A crucial indicator of blood glucose level is glycated hemoglobin (HbA1c) levels. It has been reported that every 1% increase in HbA1c levels independently predicts a 19% increase in the odds of experiencing a MI after accounting for other risk factors, including DM [5].

The prevalence of CVD in DM is a worldwide dilemma and one that occurs in both genders of all ethnicities, as suggested by several studies [6]. The INTERHEART case-control study, conducted in 52 countries throughout Europe, Asia, the Middle East, Australia, Africa, and North and South America, found nine modifiable contributors to acute MI that lead to CVD. These modifiable risk factors include hypertension, diabetes, smoking, abdominal obesity, psychological index, lack of exercise, lack of fruits and vegetables, apolipoprotein B/apolipoprotein A1 (ApoB/ApoA1) ratio, and the use of alcohol. In India, a high prevalence of metabolic cardiovascular risk factors has been reported among clinic-based patients with diabetes [7]. It is of global interest to conduct further research into this untreatable disease and how it is affecting the lives of millions.

Diabetes and CVD pathophysiology

The pathophysiology of coronary heart disease due to hyperglycemia includes the processes of atherosclerosis, oxidative stress, inflammatory changes, abnormal protein folding, increased coagulability, and disruption of the endothelial NO synthase system of the coronary endothelium which leads to endothelial dysfunction, impaired blood flow, and reduced tissue perfusion of the myocardium. Oxidative stress is the major factor that leads to MI. Another factor worth mentioning here is that fluctuations in blood glucose levels have more oxidative stress on the microvasculature than sustained hyperglycemia [8]; hence, measuring blood glucose values other than HbA1c is also necessary to evaluate the degree of diabetes and its related complications [9].

These factors are studied further in detail to examine the critical effects of uncontrolled diabetes on the micro and macrovasculature of the body.

Atherosclerosis is the major outcome of the destructive effects of hyperglycemia in diabetic patients [10]. High blood glucose causes several metabolic and structural disturbances in the blood vessels. The process by which glucose brings about these potentially destructive changes is called glycation. Glucose binds with protein residues (e.g., lysine, arginine, etc.) via this process to form Amadori complexes, of which HbA1c is a well-known example. These complexes then change to advanced glycation end-products (AGE) by oxidative, non-oxidative, and spontaneous pathways. These products react with circulating hemoglobin to form hemoglobin-AGE, and their levels are elevated in diabetic patients. AGE products bind to their receptors (RAGE) present on smooth muscles, endothelium, and cells of the immune system and bring about oxidative stress (via protein kinase C, overproduction of superoxide anion and other reactive oxygen species (ROS), arterial stiffness, atherosclerotic changes, induction of cytokines, etc.) Thus, they play a major role in degenerative diseases such as diabetes, Alzheimer’s disease, and chronic kidney diseases. Apart from this, insulin resistance itself induces pro-inflammatory cytokines such as C-reactive protein and interleukin 6 which contribute to atherosclerosis. All of these destructive changes and atherosclerotic processes that are more often seen in diabetic patients cause plaque and thrombus formation which manifests as MI [11].

Rationale

Pakistan is the sixth most populous country in the world, and DM has been a growing public health issue in Pakistan [12], with an 11.77% prevalence of diabetes [12], that is, more than 25 million people according to the current population of Pakistan. The vast majority of the population belongs to a poor socioeconomic background and low educational status. Because DM is vastly concerned with a healthy lifestyle and awareness about diabetic control measures, this research has been conducted to assess the extent of diabetes management and outcomes among diabetic patients in the study area. We also hope to create general awareness about the risk of MI in diabetic patients and guide their perceptions of it, so that necessary lifestyle changes and medical help may be provided in time.

Objectives

The primary objectives of this study were to determine an association between high HbA1c levels and fasting blood sugar levels in diabetic patients with the prevalence of MI and to compare the frequency of MI in controlled and uncontrolled diabetics. The secondary objective of the study was to find concomitant risk factors along with DM which contribute to MI. We hypothesize that there is a positive relationship between uncontrolled diabetes and the risk of MI.

## Materials and methods

Study design and study population

A hospital-based, analytical, cross-sectional study was conducted in three tertiary care hospitals, namely, Khyber Teaching Hospital, Hayatabad Medical Complex, and Peshawar Institute of Cardiology, in the city of Peshawar, KPK. These hospitals were chosen using a purposive strategy as they mainly cater to the 4 million-plus population of Peshawar and are situated in different geographical regions of the district. Moreover, such a study has not been conducted in these hospitals yet. Data were mainly collected from the Cardiology, Endocrinology, and Medical units of these hospitals. Our study population included clinically diagnosed type II diabetic patients aged 30-80 years, both males and females with or without MI. The study period was from April 2021 to March 2022 during which data were collected within two months of time, as shown by the Gantt chart inserted below (Table 1).

**Table 1:** Gantt chart.

Activities	April 2021	May 2021	June 2021	July 2021	August 2021	September 2021	October 2021	November 2021	December 2021	January–March 2022
Selecting the research topic and preparing a synopsis	X									
Ethical approval	X									
Preparing the introduction, literature review, and abstract		X	X							
Preparing the variables and questionnaire				X						
Preparing the research methodology and pilot study				X	X					
Data collection						X	X			
Data analysis								X	X	
Report writing									X	X

Sampling

The sample size of 236 was achieved using the Cochran formula where the estimated prevalence of MI in diabetics was taken to be 0.19 (19%) [5]. The z-value was taken as 1.96 and the margin of error as 0.05. A purposive sampling (judgment sampling) strategy was devised for this study. It is a type of non-probability sampling that requires researchers to have prior knowledge about the purpose of their studies so that they can properly choose and approach eligible participants for surveys. Researchers use purposive sampling when they want to access a particular subset of people as all participants of a survey are selected because they fit a particular profile. The main objective of a purposive sample is to produce a sample that can be logically assumed to be representative of the population.

The researchers visited the selected hospitals to collect data from only confirmed diabetic patients regardless of their history of MI. For patients who had an MI, only those were included who had their MI after their diabetes was diagnosed, and both good and bad glycemic control patients were included. Patients with both HbA1c more than 7% and less than 7% were purposefully selected and included in the study (maximum variation). Patients who fulfilled the inclusion criteria were selected to complete the sample size. Inclusion criteria included male and female known diabetic patients between 30 and 80 years of age who consented to participate in the study. Diabetic patients who were not willing to cooperate were excluded from our study. Moreover, patients who developed diabetes after MI were also excluded.

Procedures

The data collection tool was a structured questionnaire and an on-site survey involving live, face-to-face interviews by trained interviewers. It was also complemented with secondary data from patients’ medical records that were available for every patient included in the study. Data were recorded by the investigators themselves. The questionnaire was validated using face and content validity by CVD/diabetes and public health specialists. Second, it was reviewed after a pilot study on 10% of the calculated total sample size. Moreover, all cut-off values/ranges for variables in the questionnaire were taken with regard to the National Institute for Health and Care Excellence (NICE) UK guidelines and according to the significant effect of the variable on MI.

All values of HbA1c and other variables were recorded before the occurrence of MI in patients so that a temporal relationship could be established. HbA1c levels were taken from patient files, ranging from three months to one year before the occurrence of MI in the patients, as indicated in the questionnaire (Q14: Before the occurrence of MI, if MI occurred). For patients who did not have an MI, the HbA1c levels were recorded from three months to one year before inclusion in the study. Where multiple HbA1c tests were available during that period, a mean of the level was recorded.

The study variables included HbA1c levels​ and fasting blood glucose level​s as independent variables and MI attacks as dependent variables. Other risk factors studied included hypertension, family history of MI, body mass index (BMI), and lipid profiles. The questionnaire included questions related to the onset of diabetes and the age of occurrence of MI. It also included basic personal information (age, gender, marital status, education level, occupation type, family income, etc.) and the prevalence of behavioral risk factors (smoking, diet, physical activity, etc.). No more than 5% of the data were missing for any variable apart from lipid profile where a significant amount of data was not available in patient records. Because lipid profile was not a part of our primary objectives, it had little significance on the findings of the study and was not included in the logistical regression analysis.

Biases

To reduce the information bias, structured questionnaires were used along with secondary data from the patients’ medical files. Interviewers were the researchers themselves and were trained to conduct the surveys using local language skills and technical knowledge of the questions. To reduce the selection bias (sampling bias), purposive sampling was done on a calculated large sample that most closely represented the CVD diabetic population of Peshawar. Moreover, the study area included three major hospitals situated in different locations and covering the vast majority of the district area. Furthermore, both inpatient and outpatient data were recorded. All these steps were done to increase the external validity of the research. Finally, the effect of potential risk factors (confounding bias) was adjusted using analytical techniques such as logistical regression models, and only the significantly associated variables with MI were adjusted for.

Analysis

For analysis, we used the Statistical Package for the Social Sciences (SPSS) software version 23 (IBM Corp., Armonk, NY, USA). To determine the statistical significance of our results we performed descriptive statistics, chi-squared tests, prevalence odds ratio, and regression models, and included 95% confidence interval (CI) values as well. P-values of <0.05 were considered significant. Diabetic patients aged 30-80 years with HbA1C levels less than 7% were categorized as “controlled diabetics” and HbA1C levels of more than 7% were categorized as “uncontrolled diabetics.”

Ethics approval and consent to participate

For this study, ethical clearance was given by the Institutional Research and Ethical Review Board (IREB) of Khyber Medical College, Peshawar (vide office letter number 740/IREB/KMC dated 29-04-2021). The office letter reference number HOD/06/04 granted permission from the Ethics Committee of Peshawar Institute of Cardiology. The office letter reference number 534/HEC/B&PSC/2022 granted permission from the IREB of Hayatabad Medical Complex, Peshawar.

The confidentiality of respondents was maintained and no names or photographs were used. No forced participation in research without consent was assured. Verbal consent was taken after explaining the questionnaire-based survey and that no sensitive information was recorded. It was confirmed that no offensive questions were asked from the volunteers.

## Results

Data of 237 patients were recorded. The gender distribution was 121 (50.8%) females and 116 (48.9%) males. Overall, 82.1% of patients were obese and 17.9% were non-obese. In total, 92% of patients were non-smokers. Regarding comorbidities, 124 (52.32%) patients were hypertensive, 57 (24.05%) had no diagnosed comorbidity, 23 (9.7%) had some type of vascular disease, and 33 (13.92%) had other diseases such as respiratory, renal, neurological, and infectious diseases. Overall, 19.8% of patients had HbA1c levels of less than 7% and 80.2% of patients had HbA1c levels of more than 7%. The HbA1c levels were further stratified into different levels (Table 2). A significant association was noted between patient education and HbA1c groups (chi-square (4, N = 235) = 13.70, p = 0.008). A larger proportion of secondary school, graduate, and postgraduate patients had better glycemic control compared to the rest. This showed that education level played a key role in diabetes management.

**Table 2:** Crosstab for HbA1c levels and the prevalence of MI. The table shows the association of different levels of patients’ HbA1c levels with the prevalence of MI. A significant interaction was found between the two (chi-square (6, N = 237) = 21.40, p=0.002). HbA1c levels of 8-9% account for 47.9% (n = 46) of all MIs. HbA1c: glycated hemoglobin; MI: myocardial infarction

Patients’ HbA1C levels (before MI)	MI attacks		Total	P-value
	No	Yes		
<6%	7	0	7	0.002
	100%	0.0%		
6.0–6.4%	7	1	8	
	87.5%	12.5%		
6.5–7.0%	27	5	32	
	84.4%	15.6%		
7.0–8.0%	25	15	40	
	62.5%	37.5%		
8.0–9.0%	47	46	93	
	50.5%	49.5%		
9.0–10.0%	16	16	32	
	50.0%	50.0%		
>10.0%	12	13	25	
	48.0%	52.0%		
Total	141 (49.5%)	96 (40.5%)	237	

**Figure 1:**
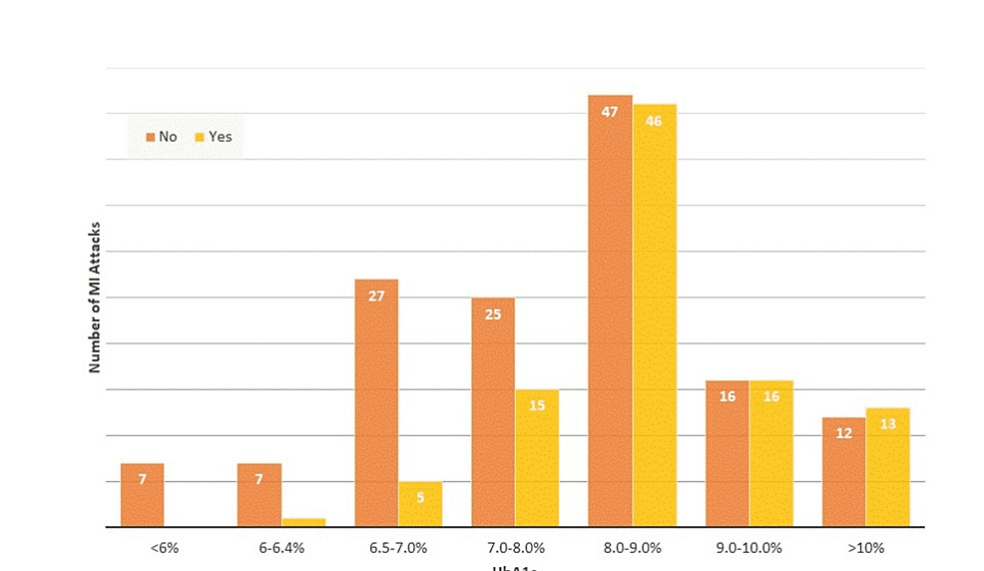
Bar chart for different levels of patients’ HbA1c with the number of MIs. HbA1c: glycated hemoglobin; MI: myocardial infarction

**Figure 2:**
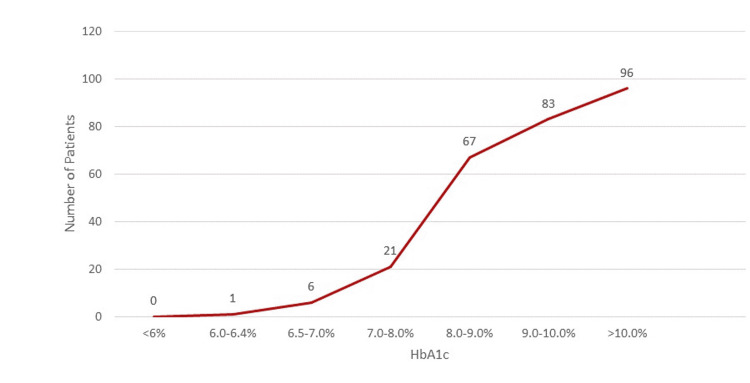
Cumulative MI attacks at different levels of HbA1c. HbA1c: glycated hemoglobin; MI: myocardial infarction

**Figure 3:**
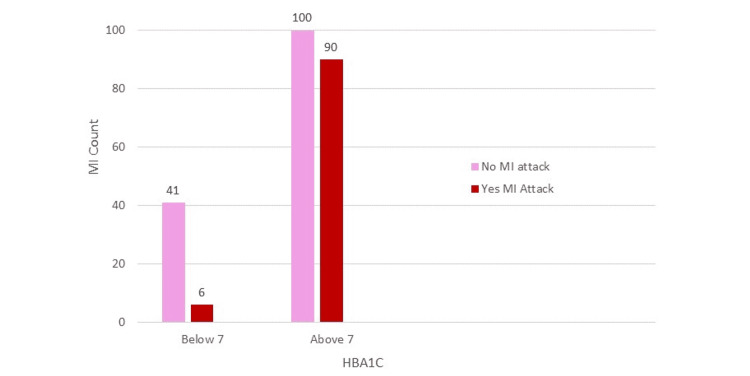
Bar chart illustrating MI attacks with HbA1c levels of more than 7% (uncontrolled diabetes) and less than 7% (controlled diabetes). HbA1c: glycated hemoglobin; MI: myocardial infarction

**Figure 4:**
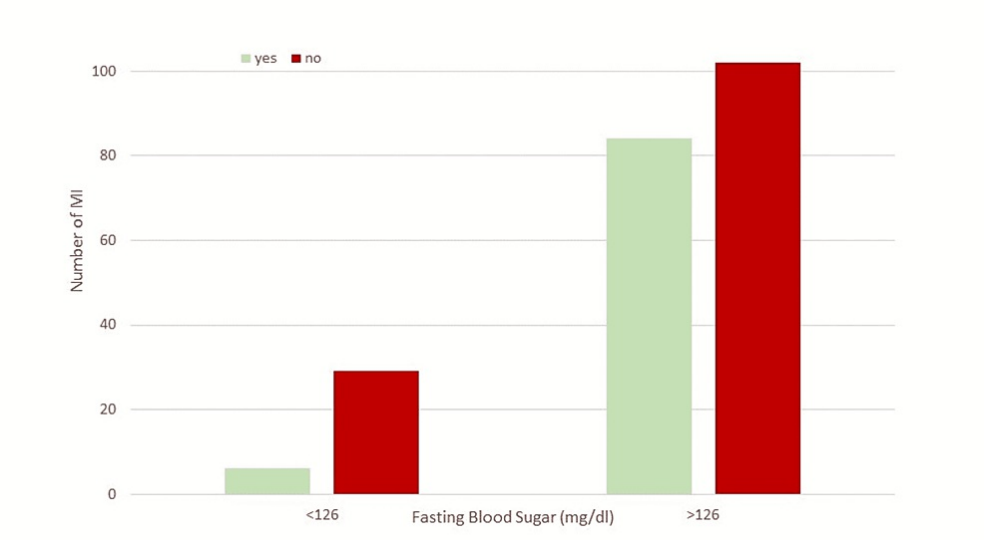
Bar chart illustrating fasting blood sugar and MI attacks. The figure shows the association between fasting blood sugar above and below 126 mg/dL with MI attacks. A significant association was found between the two (chi-square (1, N = 221) = 9.58, p = 0.002). The POR value is 3.98 showing that patients who have a fasting blood sugar level above 126 mg/dL have four times more MI attacks. HbA1c: glycated hemoglobin; MI: myocardial infarction; POR: prevalence odds ratio

The association of patients’ HbA1c levels with the prevalence of MI is depicted as a bar chart in Figure 1 and as a line graph in Figure 2. Table 3 shows the crosstab for MI attacks among patients with HbA1c of more than 7% (uncontrolled diabetes) and less than 7% (controlled diabetes); this is further illustrated as a bar chart in Figure 3. To remove the effect of other significant risk factors such as hypertension and family history, a logistical regression model was used, as shown in Table 4. Table 5 shows the prevalence of MI with each percentage increase in HbA1c level. As this study is a cross-sectional prevalence study, we calculated the prevalence odds ratio instead of the odds ratio, which is calculated in case-control and cohort studies. The association of fasting blood sugar levels with the prevalence of MI is shown in Figure 4. Table 6 shows the crosstabs of the additional risk factors with MI.

**Table 3:** Crosstab for MI attacks with HbA1c levels of more than 7% (uncontrolled diabetes) and less than 7% (controlled diabetes). The table shows the crosstabs for MI attacks of patients with HbA1c levels of more than 7% (uncontrolled diabetes) and less than 7% (controlled diabetes). The crude prevalence odds ratio was 6.15 showing that patients who have HbA1c levels above 7% have six times more prevalence of an MI attack. The prevalence rate was 40.5% for all MIs in the study population. A significant interaction was found between the two variables (chi-square (1, N = 237) = 18.72, p < 0.001). HbA1c: glycated hemoglobin; MI: myocardial infarction; POR: prevalence odds ratio; CI: confidence interval

HbA1C	MI attacks		Total	POR (95% CI)	Prevalence rate	P-value
	No	Yes				
<7%	41	6	47	6.15 (2.49–15.17)	40.5%	<0.001
>7%	100	90	190			
Total	141	96	237			

**Table 4:** Logistic regression findings. The table shows the regression model for HbA1c and the confounding variables, namely, hypertension and family history. Only the statistically significant confounding variables were used. The adjusted prevalence odds ratio for HbA1c is 6.105. HbA1c: glycated hemoglobin; MI: myocardial infarction; CI: confidence interval; SE: standard error

Logistic regression		B	SE	Wald	Sig.	Exp(B)	95% CI for Exp(B)	
							Lower	Upper
Step 1a	HbA1C	1.809	0.473	14.629	0.000	6.105	2.416	15.426
	Hypertension	0.295	0.297	0.990	0.320	1.343	0.751	2.402
	Family history	1.106	0.310	12.763	0.000	3.024	1.648	5.548

**Table 5:** Prevalence of MI with each percentage increase in HbA1c level. The table shows the prevalence of MI with each percentage increase in HbA1c levels. The mean value with the 95% confidence interval is 10.4 ± 8.1. This shows that with every 1% increase in HbA1c levels (above 6%), a 10% increase in the prevalence of MI is seen. HbA1c: glycated hemoglobin; MI: myocardial infarction; CI: confidence interval

HbA1c	Prevalence of MI with each level of HbA1c	Percentage increase (difference) for each level of HbA1c	Mean prevalence rate of MI for each percentage increase in HbA1c with 95% CI
6.0–7.0%	6/40 × 100 = 15%	15	10.4 ± 8.1
7.0–8.0%	15/40 × 100 = 37.5%	22.5	
8.0–9.0%	46/93 × 100 = 49.5%	12	
9.0–10.0%	16/32 × 100 = 50.0%	0.5	
>10.0%	13/25 × 100 = 52.0%	2	

**Table 6:** Crosstab showing the association of other variables with MI. The association of other risk factors with MI using chi-square statistics. Blood pressure and family history showed a significant association with MI. MI: myocardial infarction

Variables	MI attacks		P-value
	Yes	No	
Blood pressure (mmHg)			0.01
Normotensive (<135/<85)	80	38	
Hypertensive (>135/>85)	61	58	
Cholesterol (mg/dL)			0.823
Low cholesterol (<200)	5	10	
High cholesterol (>200)	2	5	
Body mass index			0.299
Non-obese (<30)	112	76	
Obese (>30)	28	13	
Family history of MI			<0.001
No	72	24	
Yes	69	72	

## Discussion

The main focus of our research was to highlight the prevalence of heart attacks in uncontrolled diabetes patients. There have been many studies on microvascular changes caused by poor glycemic control and the association of HbA1c with the severity of CAD but few on the occurrence of MI, especially in our study area.

A cross-sectional study was carried out in Dhaka, Bangladesh on 100 patients who were divided into two groups: HbA1c >6.5% (Group A) and HbA1c <6.5% (Group B) [13]. Group A had six times more prevalence of acute coronary syndrome (MI) than Group B. The study showed a statistically significant correlation between HbA1c and the severity of CAD (p < 0.001). Positive family history of IHD was also significantly associated with CAD (p = 0.001). All these findings conform to our findings. This study also showed a significant association between CAD incidents with dyslipidemia (p = 0.001). However, in our study, the association of MI with increased cholesterol levels was insignificant due to insufficient data concerning lipid profiles.

A study carried out in Peshawar Lady Reading Hospital in 2020 studied the degree of severity of CAD with HbA1c levels categorizing patients into two groups, namely, poor glycemic control (HbA1c >7.5%) and good glycemic control (HbA1c <7.5%) [14]. The study found a significant association between poor glycemic control and the severity of CAD. Fisher’s exact test for DM and hypertension with glycemic control showed a significant association (p = 0.005). We observed similar results in our study with blood pressure and MI (p = 0.009). In their study, the two-tailed Mann-Whitney U test was significant (p < 0.001) for age with glycemic control but insignificant for BMI. As poor glycemic control is directly related to the severe outcomes of CAD, these findings are consistent with our study findings. We observed a similar statistical association between patient age with MI (p < 0.001) and patient’s BMI with MI (p = 0.299).

A cohort study carried out in Norway involving over 4,000 patients reported that the risk of acute MI is higher in obese men but lower in overweight women [15]. In our study, 63.4% of obese patients were females; hence, the phenomenon of the obesity paradox was seen and an insignificant association was noted between BMI and MI. Many other studies have also observed the same paradoxical effect [16]. The reason for this effect is beyond the scope of our study but has been explained by others to be the presence of natural reserves to tackle the effects of pathological stress and at the molecular level about its protective effect. Other studies that disagree with this false perception report accountable factors such as the limitations of adjusting all confounding variables, the difference in the nature of comorbidities among study groups (e.g., age), and that BMI itself is not the best measure of morbid adiposities.

According to a cohort study by Selvin et al, a 1% increase in HbA1c levels increased the risk of having a cardiovascular event by 18% [17]. Our study found that with a 1% increase in HbA1c (6% and above), there was a 10.4% (95% CI = 2.3-18.5) increase in the prevalence of MI.

In our study, the association of fasting blood glucose with the prevalence of MI was statistically significant (p = 0.017). With a fasting blood glucose level of 126 mg/dL, there is a fourfold increase in MI compared to those who had a fasting blood glucose level of <126 mg/dL. Increased fasting blood glucose is a good indicator of the risk of developing cardiovascular disease and MI. Many studies have demonstrated the effect of increasing fasting blood glucose on the outcome of CAD and MI [18].

A cohort study was conducted on 1,687 patients to examine the effect of HbA1c on cardiovascular outcomes in diabetic patients with and without the presence of vascular disease [19]. The study found no statistically significant association of HbA1c with cardiovascular outcomes in patients with vascular disease but a significant association between HbA1c and cardiovascular outcomes in patients without vascular disease. In our study, only 9.7% of patients had vascular disease; hence, conclusively, our analysis showed a strong association between HbA1c and MI.

Limitations

This study had some limitations. First, the sampling technique that could be a probability/randomized method of patient selection instead of the non-probability technique used in this study. This was due to limited access to patients and reduced patient load in hospitals, pertaining to strict rules/lockdowns during the coronavirus disease 2019 pandemic. Moreover, because complete patient medical records were missing for lipid profiles, they could not be measured accurately. Third, due to the study setting being hospitals, patients with controlled diabetes were fewer in number as mostly complicated and serious patients are admitted with high blood glucose levels. Therefore, uncontrolled diabetics were more prevalent in hospitals.

We recommend a case-control study to follow this cross-sectional prevalence study where case-control groups could be formed and matching can be done. This would further endorse the findings of this study.

## Conclusions

This study was conducted in three major hospitals in Peshawar and represented a healthy proportion of the CVD diabetic population. We found a clear strong association between increased blood glucose and the prevalence of MI. A large proportion of diabetics who had poor glycemic control showed the largest proportion of MIs. Furthermore, as the HbA1c and fasting blood sugar levels increased, so did the proportion of patients who had an MI. Every 1% increase in HbA1c increased the proportion of patients having an MI by over 10%. In addition to blood glucose, we observed blood pressure and family history to significantly increase the adverse outcomes.

This study highlights the importance of glycemic control in diabetic patients and how essential it is to maintain HbA1c levels under 7% to potentially prevent a heart attack.

## Appendices

Structured questionnaire

Section A: demographic history

Q1 Patient ID

___________________________

Q2 Gender

a. Male

b. Female

Q3 What is your age?

a. 30-40

b. 40-50

c. 50-60

d. 60-70

e. 70+

Q4 Weight and height?

Weight_____________

Height_____________

Q5 Marital status

a. Married

b. Unmarried

Q6. Employment status

a. Government job

b. Private job

c. Self-employed

Q7. What is your household income per month?

a. <20,000

b. 20,000-50,000

c. 50,000-90,000

d. >100,000

Q8. What is your education level?

a. Uneducated

b. Primary school

c. Secondary school

d. Graduate

e. Post-graduate

Q9. What is your area of residence?

_______________________________

Section B: disease history

Q10. At what age diabetes was diagnosed?

a. <30

b. 30-40

c. 40-50

d. 50-60

e. 70+

Q11. The number of attacks of MI and at what age they occur? Encircle your response.

No attacks

a. 1

b. 2

c. 3

d. >3

Age ________

Q12. Do you suffer from any disease other than diabetes?

a. Hypertension

b. Thromboembolic events

c. Hypercoagulability

d. Vascular disease

e. Other________________________

Q13. When did these diseases (opted above) occur?

a. Before MI

b. After MI

Q14. What are your HbA1C blood sugar levels (before MI if applicable)?

a. <6%

b. 6-6.4%

c. 6.5-7%

d. 7-8%

e. 8-9%

f. 9-10%

g. >10%

Q15. What are your fasting blood sugar levels (before MI if applicable)?

a. <108 mg/dL

b. 108-125 mg/dL

c. 126-180 mg/dL

d. >180 mg/dL

Section C: risk factors

Q16. Do you exercise regularly?

a. Yes

b. No

If Yes, how frequently

a. At least 30 minutes for 5-6 days.

b. At least 30 minutes for 3- 4 days.

c. At least 30 minutes for 2- 3 days.

d. Less than 2 days.

Q17. Do you smoke (before MI if applicable)?

a. Yes

b. No

If Yes:

a. Regular smoker

b. Irregular

If a regular smoker, how many packs per day?_____

Q18. What was your blood pressure before MI?

**Table 7:** Blood pressure.

Category	Systole	Diastole
Normal	<135 mmHg	<85 mmHg
Stage 1	135–150 mmHg	85–95 mmHg
Stage 2	150–180 mmHg	95–110 mmHg
Severe	>180 mmHg	>110 mmHg

Q19. Do you have a family history of MI or any other cardiac abnormality in a first-degree relative?

a. No

b. Yes____________________

Q20. What is your lipid profile (Before MI, if applicable)?

**Table 8:** Lipid profile.

Parameter		
Cholesterol	<200 mg/dL	
Triglycerides	<150 mg/dL	
High-density lipoprotein cholesterol	60 mg/dL	
Low-density lipoprotein cholesterol	60–130 mg/dL	
Cholesterol/High-density lipoprotein ratio	4.0	

Q21. Do you think you are compliant with your diet plan advised by your physician?

a. Sometimes

b. Often

c. Never

d. All the time

Q22. How stressful are you (before your MI if applicable) on a scale of 1-10? Do you think it is impacting your mental health or lifestyle?

1-10: ______

a. Strongly agree

b. Agree

c. Disagree

d. Strongly disagree

Q23. How often do you check your blood glucose level?

a. Regular

b. Often

c. Sometimes

d. Rarely

e) Never

Section D: drug history

Q24. What sort of medication are you on?

a: Antidiabetic

b: Antihypertensive

c: Oral contraceptives

d: Antirheumatic

e: Other _____________________

Q25. Any recent change in dose or medication (before and after MI if applicable)

a. No

b. If Yes ______________________

Q26. Are you compliant with your medications?

a. Always

b. Often

c. Sometimes

d. Never
